# Control of cytoplasmic dynein force production and processivity by its C-terminal domain

**DOI:** 10.1038/ncomms7206

**Published:** 2015-02-11

**Authors:** Matthew P. Nicholas, Peter Höök, Sibylle Brenner, Caitlin L. Wynne, Richard B. Vallee, Arne Gennerich

**Affiliations:** 1 Department of Anatomy and Structural Biology and Gruss-Lipper Biophotonics Center, Albert Einstein College of Medicine, Bronx, New York 10461, USA; 2 Department of Pathology and Cell Biology, Columbia University College of Physicians and Surgeons, New York, New York 10032, USA

## Abstract

Cytoplasmic dynein is a microtubule motor involved in cargo transport, nuclear migration and cell division. Despite structural conservation of the dynein motor domain from yeast to higher eukaryotes, the extensively studied *S. cerevisiae* dynein behaves distinctly from mammalian dyneins, which produce far less force and travel over shorter distances. However, isolated reports of yeast-like force production by mammalian dynein have called interspecies differences into question. We report that functional differences between yeast and mammalian dynein are real and attributable to a C-terminal motor element absent in yeast, which resembles a ‘cap’ over the central pore of the mammalian dynein motor domain. Removal of this cap increases the force generation of rat dynein from 1 pN to a yeast-like 6 pN and greatly increases its travel distance. Our findings identify the CT-cap as a novel regulator of dynein function.

## Abstract (web summary)

Cytoplasmic dynein from the yeast *S. cerevisiae* behaves distinctly from mammalian dyneins, despite structural conservation. Here, Nicholas *et al*. identify a C-terminal domain in mammalian dynein that restricts force generation and travel distance, which, when removed, allows mammalian dynein to behave like its yeast counterpart.

## Graphical abstract

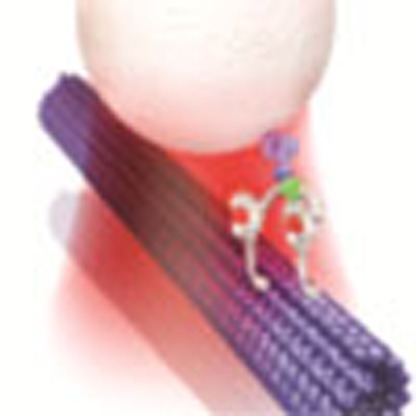

Mammalian cytoplasmic dynein plays essential roles in a wide range of both low- and high-force requiring functions during cell division, nuclear positioning and the transport of organelles and mRNAs^1^,^2^. In contrast, yeast cytoplasmic dynein is involved in a single, nonessential function, nuclear positioning^1^. Interestingly, single-molecule characterization of dynein motor behaviour across species has yielded surprisingly disparate results given the high degree of sequence and structural conservation: whereas purified native mammalian dyneins exhibit a stall force of 1–2 piconewton (pN) (refs 3, 4, 5), the extensively studied dimeric *S. cerevisiae* dynein motor domains (MDs) stall at 5–7 pN (refs 6, 7). In addition, in the absence of an opposing force, mammalian dyneins move substantially faster than yeast dynein (500 up to>1,000 nm s^−1^ versus ~100 nm s^−1^, respectively)^4^,^5^,^7^,^8^,^9^,^10^,^11^,^12^ and, under opposing force, maintain attachment to microtubules much less tenaciously (milliseconds to seconds versus tens of seconds, respectively)^4^,^6^. The basis for these striking functional differences is unknown.

Dynein is a homodimer of two identical heavy chains, each consisting of a 350–400 kDa ring-shaped MD, a slender ‘tail’ for dimerization and binding of non-catalytic subunits and accessory proteins, and a coiled-coil ‘stalk’ with an MT-binding domain (MTBD)^1^,^2^. Each dynein ring consists of six AAA+ subunits (AAA: *A*TPase *a*ssociated with diverse cellular *a*ctivities), four of which can bind and/or hydrolyse nucleotide^13^,^14^,^15^,^16^. Mechanochemical analysis has focused on dynein from *S*. *cerevisiae, Dictyostelium discoideum* and mammals. Whereas yeast and *Dictyostelium* studies have predominantly employed recombinant constructs^6^,^7^,^8^,^11^,^13^,^14^,^17^,^18^, mammalian data were until recently^19^,^20^,^21^,^22^ mostly from native dynein purified from the brain^3^,^4^,^5^,^12^,^23^,^24^ or cultured cells^22^,^25^. Intriguingly, native dynein purified from yeast and artificially dimerized yeast dynein MDs each exhibit much larger stall forces (5–7 pN versus ~1 pN)^3^,^4^,^5^,^6^,^7^,^22^ and greater processivity than dyneins from other species (~1–3 μm with runs up to ~20 μm for yeast dynein versus 300–700 nm with runs very rarely exceeding a few micrometres for mammalian dynein)^4^,^5^,^7^,^8^,^9^,^10^,^11^,^26^, though substantially slower velocity (~100 nm s^−1^ for yeast versus 500 up to >1,000 nm s^−1^ for mammalian dynein)^4^,^5^,^7^,^8^,^9^,^10^,^11^,^12^. These differences may reflect the range of roles for higher eukaryotic dyneins versus the limited physiological function for dynein in *S*. *cerevisiae*, which serves only to move the nucleus into the bud neck during mitosis^1^. However, isolated reports of yeast-like force production by porcine dynein^23^,^27^ have highlighted the need to better understand dynein force-generating behaviour. Whether differences in dynein function between yeast and mammals (and possibly among mammals) reflect evolutionary variation, protein preparation, experimental conditions or undefined features of dynein mechanical regulation remains a mystery.

Yeast and mammalian dynein motor domains exhibit a high degree of sequence and structural conservation. The only outstanding difference is the presence of a 32 kDa C-terminal motor element in mammals (also present in *Dictyostelium* and other organisms; Fig. 1a,b). This element lies flat over the dynein ring, partially occluding the central pore, and is attached by a flexible, structurally disordered ~20 amino acid (a.a.) predicted ‘hinge’^14^,^28^,^29^ to a short helix (H1) emerging from AAA6 (Fig. 1c). The C-terminal extension, referred to here as the CT-cap, also partially covers AAA1, the principle site of ATP hydrolysis (Fig. 1c and inset of Fig. 1c)^15^. Prior work revealed that the CT-cap can be separated from the rat MD by limited proteolysis, an effect inhibited by transition state ATP analogues^29^. Thus, the CT-cap might be structurally independent and capable of shifting position during the mechanochemical cycle^29^. In *Dictyostelium*, removal of the CT-cap or decreasing the flexibility of the hinge have each been reported to decrease processivity^11^. Removal of the entire C-terminal region (CT-cap plus H1) disrupted allosteric communication between the MD and MT-binding domains^14^. With this knowledge, we hypothesized that the CT-cap might be responsible for some of the functional differences between yeast and mammalian cytoplasmic dynein.

In this study, we report the first structure-function and single-molecule force measurements of a recombinant dimeric rat dynein motor domain construct. We find that functional differences between yeast and mammalian dynein are real and attributable to a C-terminal motor element absent in yeast, which, when present, resembles a ‘cap’ over the central pore of the dynein motor domain. Removal of this element increases force generation of rat dynein from 1 pN to a yeast-like 6 pN and greatly increases travel distance. Our findings identify the CT-cap as the first motor protein element responsible for controlling force production. The CT-cap potentially represents a novel locus for dynein regulation.

## Results

### Expression of motor domains with and without the CT-cap

**Figure 1:**
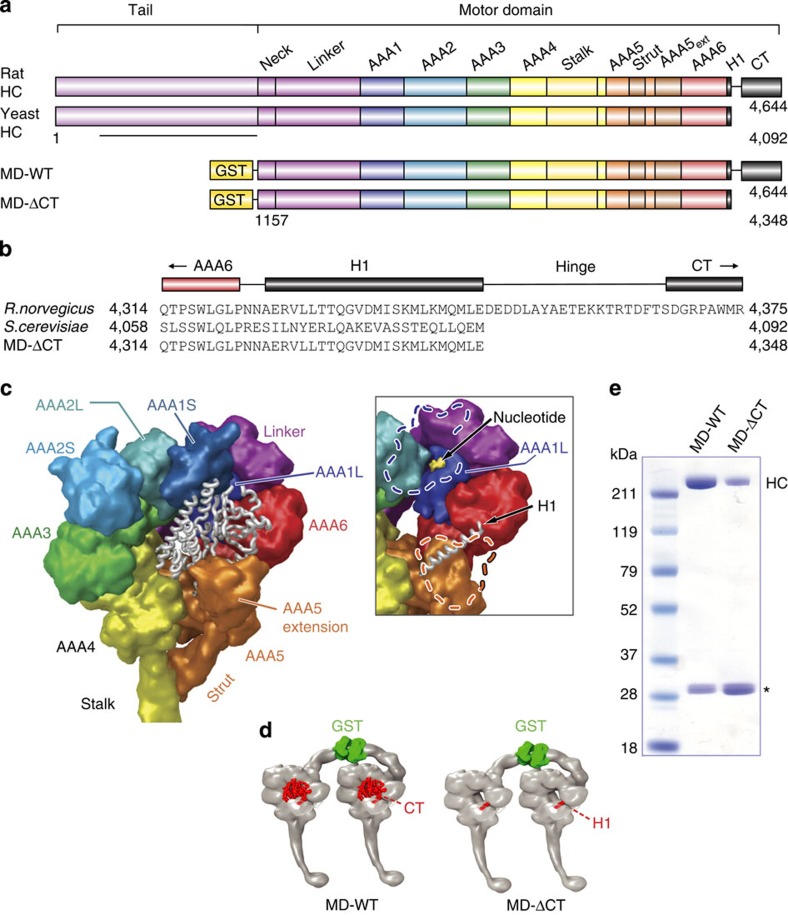
Rat cytoplasmic dynein motor domain constructs. (**a**) Domain organization of the native dynein heavy chain (HC) and the engineered constructs, MD-WT (a.a. 1,157–4,644) and MD-ΔCT (a.a. 1,157–4,348). The line indicates the HC dimerization region, which is truncated in MD-WT and MD-ΔCT and replaced with an N-terminal GST for MD dimerization. (**b**) Sequence alignment of the proximal C-termini of native rat dynein, native yeast dynein and MD-ΔCT. The MD-ΔCT truncation eliminates the hinge region and distal C-terminus, but preserves the proximal H1 helix, as in yeast. (**c**) Dynein MD structure (PDB entry 3VKH^14^). The C-terminal elements are represented as tubes in white. ‘L’ and ‘S’ indicate large and small subdomains, respectively, of AAA1 and AAA2. Inset: same view, with AAA1S, the AAA5 extension, and the CT-cap removed. The dashed outlines indicate the positions of AAA1S and the AAA5 extension. Note the AAA1 active site (formed at the interface of AAA1L, AAA1S and AAA2L) and the H1 helix (running between AAA5/AAA6 and the AAA5 extension). (**d**) Schematic illustrations of the MD-WT and MD-ΔCT constructs, created using PDB entries 3VKH^14^ and 1VF4 (see Methods section for additional information). (**e**) Coomassie-stained gel of MD-WT and MD-ΔCT purified via SpinTrap column. HC: dynein heavy chain; *free GST (see Supplementary Information).

To test our hypothesis, we generated recombinant, baculovirus-expressed dynein MDs from rat. We engineered two glutathione-*S*-transferase (GST) fusion constructs (Fig. 1a,b): one corresponding to the wild-type rat dynein (MD-WT) and the other lacking the C-terminal region (MD-ΔCT) and corresponding to *S*. *cerevisiae* dynein (Fig. 1b). Both constructs omit the dynein tail, but retain the ‘neck’ domain that connects the MD and tail and interacts with the AAA ring in a nucleotide-dependent manner^30^. Although the tail regulates motor activity^5^,^10^, it is nonessential for production of motion and force^6^,^7^,^8^,^11^,^17^,^18^,^19^.

We purified MD-WT and MD-ΔCT using glutathione affinity chromatography (Fig. 1e) (followed by size-exclusion chromatography (SEC) for some preparations; Supplementary Fig. 1a). Each construct ran as a single coherent peak by sucrose density gradient centrifugation with no evidence of aggregation (Supplementary Fig. 1a), and exhibited basal and MT-stimulated ATP hydrolysis (Supplementary Fig. 2 and Supplementary Table 1).

### MD-WT motility and force generation

**Figure 2:**
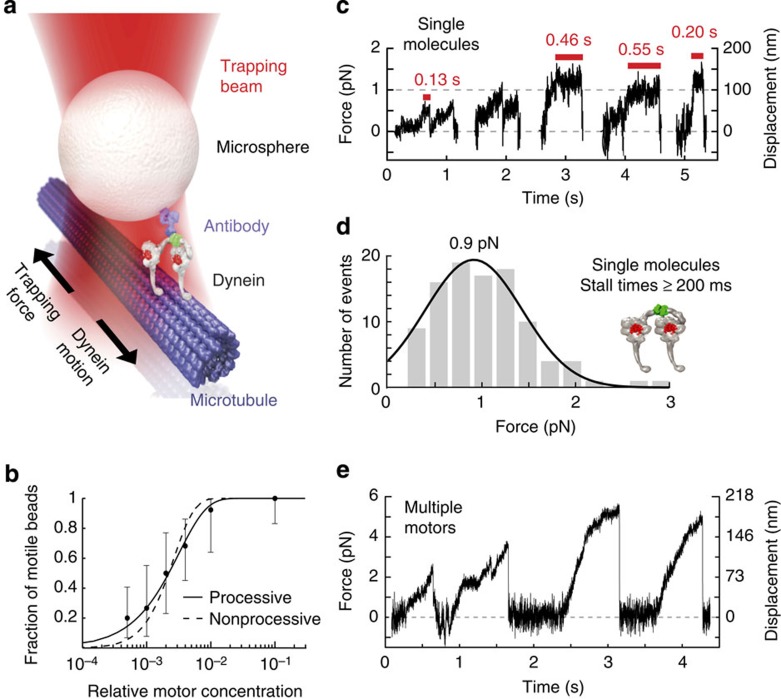
Single-molecule function of MD-WT. (**a**) Illustration of the optical trapping assay. GST-dynein is attached via an anti-GST antibody to a 1- μm polystyrene microsphere (‘bead’) that is optically trapped above a MT. As dynein moves along the MT, the trap exerts an opposing force. (**b**) Fraction of motile beads (those generating forces ≥0.5 pN using a trap stiffness of *k*=0.01 pN nm^−1^) versus the relative MD-WT concentration. Error bars are Clopper–Pearson 95% confidence intervals (95% CIs) of the mean. We tested 14–25 beads at each concentration (109 total). The curves are fits assuming processive (solid line) or nonprocessive (dashed line) motors (see Methods). The data are best fit by the processive model (coefficient of determination *R*^2^=0.99 versus *R*^2^=0.91; *F*-test *P*-value=0.02). (**c**) Representative examples of MD-WT force generation (1 mM ATP) at motor concentrations for which 50% or fewer beads moved. Red bars indicate duration of maximal sustained force. (**d**) Histogram of stall forces (maximal forces sustained for≥200 ms), with average 1.0±0.5 pN (mean±s.d.). The curve is a Gaussian fit to the data (mean 0.9 pN and s.d. 0.5 pN; 95% CIs (0.8, 1.0) and (0.4, 0.6) pN, respectively). Of 381 MT encounters (derived from 54 beads over 19 experiments), 100 (26%) met the criterion for stalling. (**e**) Example of large forces produced by MD-WT at high motor concentration (100% fraction of motile beads). Experiments were performed with AC-purified protein (**e**), with AC-/SEC-purified protein (**b**,**c**), and with both AC-purified and AC-/SEC-purified protein (**d**).

Coverslips coated with MD-WT supported robust MT gliding at up to ~460 nm s^−1^ (Supplementary Movie 1 and Supplementary Fig. 3), demonstrating clear multi-motor motility. To study MD-WT single-molecule function, we used optical tweezers (Fig. 2a) as described previously^31^. Analysis of the fraction of motile beads as a function of motor concentration (Fig. 2b) showed that single MD-WT particles move and produce forces ≥0.5 pN (~50 nm displacement for a trap stiffness of *k*≈0.01 pN nm^−1^).

At single-molecule concentrations (≤50% beads exhibiting motion), MD-WT exhibited a range of motile behaviour (Fig. 2c and Supplementary Fig. 4). The motor often detached after several steps, and sometimes stalled, with most runs lasting ≤2–3 s and with velocities often exceeding 200 nm s^−1^. Similar to criteria used previously^5^, we defined stalling as the maximum force achieved and sustained for at least 200 ms during a single-MT encounter (26% of motile events). The MD-WT distribution of stall-forces showed a single peak at 0.9±0.5 pN (mean±s.d.) (Fig. 2d). Maximal forces (irrespective of duration) were similar, but the distribution was skewed slightly towards smaller forces (Supplementary Fig. 5), indicative of premature MT detachments before stalling^5^ (average run length ~90±50 nm for *k*≈0.01 pN nm^−1^). Prolonged stalling of ≥0.5 s was rare (~9% of events).

Thus, MD-WT produces forces similar to those reported for the complete mammalian cytoplasmic dynein complex^3^,^4^,^5^, but is quite distinct from GST-dimerized yeast dyneins, which are highly processive and stall at ~4–5 pN for tens of seconds^6^,^7^. We saw no evidence of the ~7 pN stalling reported in two previous wilde-type mammalian dynein studies^23^,^27^. However, at high MD-WT concentrations (several times that needed for 100% bead movement), we observed maximal forces of ~5–7 pN (and occasionally greater) and long runs (≥200 nm) (Fig. 2e), probably attributable to the action of multiple MD-WT molecules. Surprisingly, these beads also occasionally moved bidirectionally (Supplementary Fig. 6), which we never observed at single-molecule concentrations. The basis for these effects is unclear and warrants future investigation (see Supplementary Information for an extended discussion).

### MD-ΔCT motility and force generation

**Figure 3:**
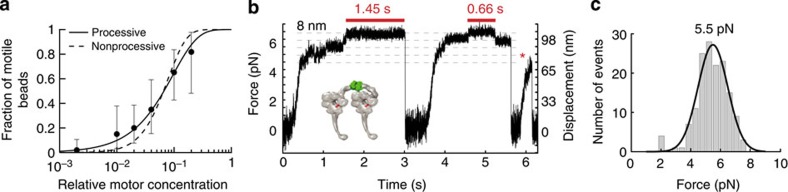
Force generation of single MD-ΔCT molecules. (**a**) Processivity analysis, as in Fig. 2b. The data are best fit by the processive model (*R*^2^=0.99 versus *R*^2^=0.89; *F*-test *P*-value=0.01). We tested 11–50 beads at each concentration (154 total). (**b**) Example of single-motor force generation (*k=*0.061 pN nm^−1^). Steps are visible after reaching ~4 pN. The two red bars indicate periods of stalling (here at ~6.5 pN). The red asterisk marks a run terminated by detachment before stalling. (**c**) Histogram of stall forces (maximal forces sustained for ≥400 ms), with average 5.4±1.1 pN (mean±s.d.) and Gaussian fit (mean 5.5 pN; s.d. 1.0 pN; 95% CI’s (5.3, 5.7) and (0.8, 1.2) pN, respectively). Of 340 total MT encounters (derived from 25 beads over 8 separate experiments), 135 (44%) met the stalling criterion. ATP concentration was 1 mM. Experiments were performed with AC-purified protein (**a**,**b**) and with both AC-purified and AC-/SEC-purified protein (**c**).

**Figure 4:**
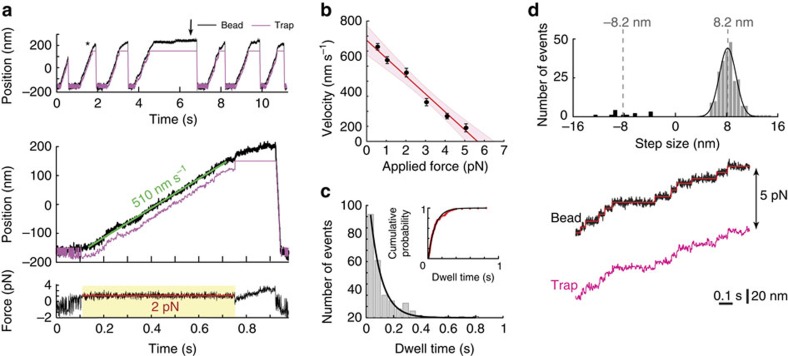
Single-molecule function of MD-ΔCT. (**a**) Bead movement under constant load (optical trap force clamp). Top: repeated bead displacements by single motors (black). The trap (magenta) follows at a fixed distance to apply a constant force (here, 2 pN). When the bead travels beyond the region of force-clamp operation, it either stalls (black arrow) or detaches. Bottom: detail of the event marked by an asterisk in the top panel, fit with a line to measure the mean velocity (~510 nm s^−1^). The lower inset shows the applied force, which was held constant at 2.1±0.3 pN (mean±s.d.) during force-clamp operation (yellow region). (**b**) Velocity versus force. Points are means of repeated measurements under constant force, including only runs ≥50 nm. Error bars span 95% CIs of the mean. The red line is a weighted linear fit (*V*=672 nm s^−1^−119 nm s^−1^ pN^−1^ × *F*), with shaded region of 95% confidence that intercepts the abscissa at (575, 769) nm s^−1^ and the ordinate at (5.1, 6.5) pN. Data are from six beads over two experiments (*N*=38–233 at each force; 655 events total). (**c**) Histogram of dwell times between consecutive forward steps under 5 pN load. An exponential fit, *y*=*A* exp(−*k*_cat_ *t*), gives *k*_cat_ =12.1 s^−1^; 95% CI (10.2, 13.9) s^−1^. Inset: empirical cumulative probability density function, with fit *y*=1−exp(−*k*_cat_ (*t*−*t*_L_)), where *t*_L_ is the dwell time detection limit (~6–8 ms) and *k*_cat_ =10.6 s^−1^ (95% CI (10.4, 10.8) s^−1^). Data from four beads over three separate experiments (211 events). (**d**) Top: histogram of step sizes under 5 pN load (*N*=254). Grey and black bars represent forward (95% of steps) and backward steps, respectively. A Gaussian fit (black curve) to the forward steps yields 8.2±1.3 nm (mean±s.d.; 95% CI’s (8.0, 8.4) and (1.1, 1.5) nm, respectively). Backward steps were 7.8±2.7 nm (mean±s.d.). Bottom: example trace showing steps (red line) identified by a step-finding algorithm (see Methods). ATP concentration was 1 mM. Experiments were performed with AC-purified protein.

We next analysed MD-ΔCT, which lacks the CT-cap region. Motility was strikingly different from that of MD-WT. Ensembles of MD-ΔCT did not glide MTs (Supplementary Movies 2 and 3) or undergo ATP-induced MT dissociation (Supplementary Fig. 7b; see also Supplementary Information for an extended discussion). However, compared with MD-WT, MD-ΔCT was much more processive and exhibited robust motility and prolonged runs at the single-molecule level in the optical trap (Figs 3a,b and 4a). It tenaciously stalled at 5.5±1.0 pN (mean±s.d.) (Fig. 3c), comparable to the behaviour of the analogous yeast dynein construct^6^,^7^. To study the MD-ΔCT construct under a constant applied load, we used feedback-based force-clamp experiments in which the optical trap follows bead motion at a fixed distance. MD-ΔCT routinely moved over 400–500 nm (Fig. 4a), even under loads approaching its stall force (in contrast to MD-WT, for which similar experiments were not feasible due to short run lengths). Velocity-versus-force analysis (Fig. 4b) predicted stalling at 5.7 pN (95% CI (5.1, 6.5) pN), in agreement with our initial observations (Fig. 3b,c). These findings demonstrate the CT-cap to be a potent regulator of dynein force output and processivity, and suggest that its absence accounts for the greater force generation of yeast dynein.

MD-ΔCT did exhibit some notable differences from the analogous WT yeast dynein constructs. First, MD-ΔCT retains the higher velocity of mammalian dyneins, projected to reach ~670 nm s^−1^ in the absence of opposing load (intercept with the abscissa in Fig. 4b; Supplementary Fig. 8). Second, MD-ΔCT steps are short and consistent, with few backward steps. Under 5-pN load, the distribution of dwell times between steps is well described by a single exponential (rate constant *k*_cat_ ≈11–12 s^−1^) (Fig. 4c). At this force, MD-ΔCT moves at ~90 nm s^−1^ (95% CI (64, 117) nm s^−1^) (Fig. 4b), implying ~8 nm steps if step size is constant. Direct analysis of step sizes confirms a narrow distribution of 8.2±1.3 nm (mean±s.d.) with very few (5%) backward steps (Fig. 4d), similar to kinesin^32^. Resolving steps at low load (0.5 pN force clamp with 5 μM ATP) is difficult, but we saw no evidence of increased average step size at low load (Supplementary Fig. 9), in contrast to an earlier study of native mammalian dynein^3^. The Michaelis–Menten kinetics of velocity observed at 0.5-pN load suggests that step size is also independent of ATP concentration (Supplementary Fig. 8). The stepping behaviour just described differs from that we and others have reported for analogous yeast dynein constructs both in the absence^8^,^17^,^18^ and presence^6^ of force. These studies also reported a broader distribution of step sizes, more frequent backward steps, and near the stall force, more 4-nm steps and the emergence of ‘non-advancing’ stepping characterized by repeated forward-backward displacements^6^. Taken together, the differences in velocity and stepping behaviour between yeast dynein and MD-ΔCT demonstrate that, in addition to the CT-cap, other more subtle variations within the MD may also contribute to interspecies differences between dyneins.

## Discussion

Here, using combined structure-function and single-molecule force measurements on a recombinant GST-dimerized rat dynein, we demonstrate that functional differences between yeast and mammalian dynein are primarily attributable to the relatively little explored C-terminal motor domain structure, which is absent in yeast and present in mammalian dynein as a ‘cap’ over the central pore of the motor domain. Removal of the CT-cap imparts rat dynein with increased processivity and yeast dynein-like force-generation capabilities. We speculate that the CT-cap may act as a target for regulatory factors and/or post-translational modifications responsible for modulating mammalian dynein processivity and force output for dynein’s numerous and diverse cellular functions. For example, modification of the CT-cap could help fine-tune dynein’s force generation for high-load functions, such as nucleokinesis, and low-load functions, including the long-distance transport of mRNA and vesicles.

The underlying molecular mechanism by which the CT-cap regulates dynein force production and processivity now emerges as an important question in the field. Intriguingly, the CT-cap lies over the ATPase cleft of AAA1-AAA2 (ref. 14), suggesting a potential role in regulating nucleotide access to dynein’s main active site. Proteolytic removal of the CT-cap is sensitive to nucleotide occupancy of this site, and more immediately significant, ATPase activity is reduced and the *K*_i_ for vanadate markedly increased on the removal of the CT-cap^29^. Crystallographic analysis revealed residual nucleotide in AAA1 for the *Dictyostelium* MD, but not yeast^13^,^14^, consistent with a decrease in affinity for ATP. Thus, the CT-cap may operate as a shutter to open or close the AAA1-AAA2 ATPase cleft, with the effect of destabilizing or stabilizing ATP/ADP binding. Nucleotide may exchange more freely in the absence of the CT-cap, resulting in reduced ATP and/or ADP affinities and prolongation of the apo MT-binding state. Extending or shortening the duration of specific steps in the mechanochemical cycle could affect force-bearing states of the dynein cross-bridge cycle and consequently increase the motor’s stall force and processivity. Whether such changes prove valid remains to be tested. In addition, the CT-cap may affect allosteric communication between the AAA ring and MTBD, as shown for a complete C-terminal truncation of the *Dictyostelium* MD (that is, CT-cap plus H1 (ref. 14); see Supplementary Information for an extended discussion).

Our findings reveal that the removal of the CT-cap increases the processivity of dynein. Whereas the MD-WT motor moves on average over ~90 nm under loads up to 0.5 pN, MD-ΔCT movement routinely exceeds the 400 nm detection range of our microscope, even under loads of up to 5 pN. These data provide the first evidence that dynein processivity can be controlled by elements within the motor itself. This is in contrast to the finding that a truncation of the *Dictyostelium* dynein C-terminus resulted in a non-processive motor^11^, perhaps attributable to the use of different termination sites (the *Dictyostelium* truncation site was distal to the one employed here and to the C-terminal boundary in yeast). Dynactin has long been known for its role in increasing dynein processivity by approximately twofold^33^, an effect recently shown to be mediated by a dynein binding, coiled-coiled element in the p150^*Glued*^ subunit of dynactin^9^,^34^. Recent work has also demonstrated that the dynactin-cargo adapter protein BicD2 markedly enhances dynein processivity by stabilizing the dynein–dynactin complex, thereby converting mammalian dynein into an ultra processive motor, which moves over distances of up to ~10 μm (refs 20, 21). The relationship between such factors, and dynactin- and CT-cap-regulated processivity remains to be addressed in detail.

The enhanced processivity of the MD-ΔCT construct allowed us to determine its force–velocity (*F–V*) relationship using the force-clamp mode of our optical tweezers. Recent work has shown that the shape of the *F–V* curve provides insight into the motor’s ability to work cooperatively in multi-motor assemblies^35^,^36^. We note that the measured *F–V* curve for the MD-ΔCT construct (Fig. 4b) differs in shape from the *F–V* curve reported for full-length yeast dynein^6^. While the *F–V* curve of yeast dynein is sigmoidal, the *F–V* curve of the MD-ΔCT motor can be approximated by a linear function. Different *F–V* curves can be expected considering that yeast dynein shows force-dependent stepping behaviour, while MD-ΔCT exhibits a unitary, load-independent step size. However, given the experimental uncertainty of our measurements at low loads (<2 pN) and high loads (>4 pN), we are hesitant to conclude which underlying differences between the motors account for the *F–V* curves.

Interestingly, the MD-ΔCT motor differs in stepping behaviour from that reported for higher eukaryotic full-length dyneins. The ~6 pN-generating MD-ΔCT construct displayed a unitary 8-nm step size independent of load, in contrast to the load-dependent stepping behaviour reported for ~1 pN-generating mammalian dynein^3^,^37^. Because full-length yeast dynein and the GST-dimerized yeast dynein construct analogous to our MD-ΔCT motor have similar stepping behaviours^6^, it is unlikely that the truncation and GST-dimerization of MD-ΔCT account for the difference. The possibility that the CT-cap can alter stepping behaviour, in addition to its impact on force generation and processivity, requires further investigation. Indeed, while full-length and truncated GST-dimerized yeast motors show load-dependent stepping behaviour, their predominant advancing step size is 8 nm even at low load^6^, as is the case for the MD-ΔCT motor. Thus, it is possible that the presence of the CT-cap in mammalian dynein affects the forward displacement of the trailing head. Presumably, the CT-cap of one motor domain is sandwiched between both motor domains, and could therefore influence the path of the rear head as it moves forward (for example, by sterically affecting head passing and/or linker element movements), biasing the motor towards larger steps under low load.

In conclusion, we have elucidated a novel role for the relatively little explored dynein CT-cap and explain previously puzzling differences between yeast and mammalian dynein. It remains to be seen how this domain exerts its effects. Future studies promise exciting insights into the mechanisms by which the CT-cap regulates the dynein nanomachine.

## Methods

### Construct design

Two dynein MD fragments, one of 399 kDa (MD-WT; a.a. 1157–4644) encompassing the entire MD and another of 367 kDa (MD-ΔCT; a.a. 1157–4348) lacking the CT-cap, were cloned from the full-length rat cytoplasmic dynein heavy chain and produced by the baculovirus expression system. The MD fragments were N-terminally fused with an in-frame GST tag for rapid purification and MD dimerization. The proper C-terminal boundary of MD-ΔCT was determined from primary sequence alignment and X-ray crystallography structural data of *D. discoideum*^14^,^28^ and *S. cerevisiae*^13^,^38^ dynein.

### Dynein expression and purification

Sf9 insect cells were infected with recombinant baculovirus for 50–55 h. The cells were washed in PBS, and recombinant dynein was extracted by homogenization in DEB (100 mM Pipes, pH 7.2, 2 mM MgCl_2_, 2 mM EGTA, 50 mM NaCl, 1 M glycerol, 0.1 mM ATP, 1 mM DTT, protease inhibitor cocktail (Sigma)). The cytosolic extract was cleared by centrifugation at 5,000 *g* for 10 min and 100,000 *g* for 30 min. The supernatant was applied to a GST SpinTrap purification column (GE Healthcare) and incubated for 20 min at 4 °C. Unbound material was removed by washing the column two times with DEB, and bound protein was eluted with 10 mM glutathione in DB (30 mM Pipes, pH 7.2, 2 mM MgCl_2_, 2 mM EGTA, 50 mM NaCl, 1 M glycerol, 1 mM DTT). In some preparations, free GST was removed by SEC fractionation on a gravity flow column manually packed with 2 ml Sephacryl S-200 (GE Heathcare) in DB (removal of free GST from MD-WT or MD-ΔCT samples by SEC did not affect the force-generation capabilities of the motor constructs, as judged by the consistent motor behaviour before and after the additional purification step). Protein concentrations, which varied between 0.1–0.2 mg ml^−1^ depending on the prep, were determined by the Bradford method, using albumin as a standard, and by densitometric analysis of band intensities on a coomassie-stained sodium dodecyl sulfate–PAGE (SDS–PAGE) gel. Purified dynein was assayed for enzymatic activity within 1 day. For single-molecule analyses, small aliquots of protein were immediately distributed to thin-walled PCR tubes, flash-frozen in liquid nitrogen and stored at −80 °C pending use.

### Sucrose gradient density ultracentrifugation

For sucrose gradient analysis, purified MD-WT and MD-ΔCT were loaded onto a 1.3-ml linear 5–20% sucrose gradients in DB buffer (30 mM Pipes, pH 7.2, 2 mM MgSO4, 2 mM EGTA, 50 mM NaCl, 1 M glycerol, 1 mM DTT). The gradients were centrifuged at 54,000 r.p.m. for 3 h at 4 °C in a Beckman TLS-55 rotor. Fractions were collected and analysed by SDS–PAGE and western blot using a monoclonal GST antibody (1E5, Santa Cruz Biotech, 1:1,000).

### Optical trapping

Optical trapping studies were performed essentially as described previously in detail^31^,^39^. Cy3-labelled MTs were bound covalently to amino-silanated glass coverslips, which were then used to make flow chambers. After appropriate dilution in assay buffer (30 mM PIPES, 2 mM MgSO_4_, 2 mM EGTA, 7.3% glycerol, 10 μM taxol, pH 7.2) with 1 mg ml^−1^ β-casein, dynein was bound to 1-μm diameter polystyrene microspheres covalently bound to anti-GST antibodies. The mixture was then supplemented with ATP (1 mM), DTT (10 mM), a pyranose oxidase-based oxygen scavenger system^40^ (25 mM glucose, 3 U ml^−1^ pyranose oxidase and 90 U ml^−1^ catalase) and β-casein (1 mg ml^−1^), and flowed into the slide chamber. In some experiments (including all experiments studying titration of ATP), an ATP regeneration system was also added (1 mM phosphoenolpyruvate, 0.1 mg ml^−1^ pyruvate kinase). The optical trapping microscope, including the automated force clamp, was controlled using software custom-written in LABVIEW (National Instruments). Data were acquired at 3 kHz after low-pass filtering at 1.5 kHz. Beads were tested for at least 4 min each to determine the presence of active motors. For analysis of single-molecule behaviour, data were considered only if 50% or fewer beads from the given experiment exhibited motility^41^, which was achieved by diluting the purified MD-WT and MD-ΔCT motor constructs 100–500 × and 10–100 × , respectively, in the assay buffer. Data visualization and analysis were performed using software custom-written in MATLAB (The MathWorks).

### Processivity analysis

We defined beads as motile if they generated ≥0.5 pN at least once during a 4 min observation period (generally, *k*=0.01 pN nm^−1^ for MD-WT or *k*=0.04–0.06 pN nm^−1^ for MD-ΔCT, with spring constants chosen such that stalling usually occurred at ~100–200 nm bead displacement). We then plotted the motile fraction versus relative motor concentration and calculated the uncertainty in each measurement (which constitutes sampling from a binomial population, that is, motile beads and non-motile beads), as the Clopper-Pearson 95% CI of the mean. We fit two models^42^ (weighted by the uncertainty in each measurement) to the data. The first, ‘processive’ model assumes one or more motors are required to move a bead: *F*=1−exp(−*λC*), where *F* is the fraction of moving beads, *C* is the relative motor concentration and *λ* is a fitting parameter that depends on the fraction of active motors. The second, ‘nonprocessive’ model assumes two or more motors are required: *F*=1−exp(−*λC*)−(*λC)* exp(−*λC*). We determined which model fit best by considering the coefficient of determination (*R*^2^) for each fit and a one-parameter *F*-test at significance level *α*=0.05.

### Stall force analysis

The stalling criterion for MD-WT was chosen empirically and based on previous work^5^. For MD-ΔCT, which robustly maintains attachment to the MT, and continues to advance slowly as it approaches stall force, we employed a somewhat more systematic approach to ensure that stalling events excluded detachment before reaching the true maximal force. We first picked candidate stalling events of ~200 ms or longer. On this data set, we performed a Lillefors goodness-of-fit test of composite normality at the *α*=0.05 significance level, under the null hypothesis that the stall forces were normally distributed (with unspecified mean and standard deviation). The test initially rejected the null hypothesis, suggesting a non-normal distribution of data. This is unexpected for true stalling events, and likely indicates that some events represented detachment from the MT before stalling occurred. We then applied a new threshold, picking only events lasting ≥250 ms, and repeated the test, again rejecting the null hypothesis. Repeating this procedure again for 300 or 400 ms threshold failed to reject the null hypothesis. We then used 400 ms as our stall-time criterion. The difference in the calculated mean for either case was small (5.3 pN for 200 ms threshold versus 5.5 pN for 400 ms threshold, or a 4% change).

### Force-clamp experiments

In custom software that controls our microscope, we implemented a simple force-clamp (force-feedback) algorithm with a proportional gain response to offsets from the force set-point. Depending on the motor velocity, we employed a feedback rate of 50–600 Hz (higher rates are required for greater velocities). During these experiments, we periodically turned off the force feedback and confirmed that the stalling behaviour was unaffected. This was done to rule out damage due to prolonged application of the constant loads. To determine average velocities for velocity versus force and Michaelis–Menten plots, we fit lines to runs ≥50 nm in length, and computed the average of all measurements (line fits) and then the 95% CI as 1.96 × s.e.m. Linear and nonlinear curve fitting was performed in MATLAB (The MathWorks) or R ( www.R-project.org), and 95% CIs for the parameters were calculated with built-in functions (with Bonferroni correction for simultaneous parameter estimation, where appropriate). For dwell time and step size analysis, the locations and sizes of steps were detected using an automatic detection algorithm^43^, as described previously^6^.

### Basal and microtubule-stimulated ATPase activities

MD-WT and MD-ΔCT were enzymatically tested for steady-state ATPase activity using the Malachite Green assay. Dynein activity was assayed at 25 °C for 30 min in DAB (30 mM Pipes, 2 mM MgCl_2_, 2 mM EGTA, 7.3% glycerol, 1 mM DTT, pH 7.2) supplemented with 0–5 mM ATP and in the absence and presence of 0.1–20 μM taxol-stabilized microtubules (tubulin; Cytoskeleton). Control samples of microtubules alone were included in the analysis. Computed ATPase activities were corrected for the presence of free GST by determining total amount of protein and the fraction of free GST by densitometric analysis of band intensities on a coomassie-stained SDS–PAGE gel.

### Microtubule gliding

Coverslips (Zeiss) were cleaned using the HCl-Ethanol protocol from the Salmon lab ( http://labs.bio.unc.edu/Salmon/protocolscoverslippreps.html). In short, coverslips were submerged in 1 M HCl for 16 h at 60 °C, followed by intensive rinsing with ddH_2_O and threefold sonication in ddH_2_O for 30 min. Subsequently, the coverslips were sonicated sequentially for 30 min each in ethanol solutions of 50% (V/V), 75% (V/V) and 95% (V/V), respectively. The coverslips were stored in 200-proof ethanol and flamed immediately before use.

MT-gliding assays were performed on a total internal reflection fluorescence (TIRF) microscope (modified Nikon Eclipse Ti) with a × 100, 1.45 NA oil immersion objective (Nikon) and a 532-nm laser (Coherent) to excite TRITC (tetramethyl rhodamine isothiocyanate)-labelled MTs (Cytoskeleton). Images were obtained using μManager^44^ and an EMCCD (electron multiplier CCD; Andor iXon Ultra) with an acquisition time of 1,000 ms. The assay buffer consisted of 30 mM PIPES, 2 mM EGTA, 2 mM MgSO_4_, 7.3% glycerol, 1 mM DTT, 20 μM taxol, pH 7.2. Rat brain dynein was attached to the coverslip by non-specific binding; rat GST dynein was linked to the coverslip via an anti-GST antibody (Abcam ab6613, diluted 1 in 10 in dynein buffer and bound non-specifically to the coverslip). Next, the coverslip surface was blocked with 2 mg ml^−1^ BSA in assay buffer. Finally, the motility solution containing 2 mg ml^−1^ BSA and varying concentrations of ATP, an ATP-regeneration system (see above), an oxygen scavenger system (see above) and 0.05 mg ml^−1^ TRITC-MTs was added. Data were analysed using ImageJ^45^,^46^ (in conjunction with the plugin MTrackJ^47^) and the data analysis software Prism (GraphPad Software).

### Dynein-MT co-sedimentation and release

Fifty microlitres of purified dynein (concentration 100 to 350 ng μl^−1^, depending on the MD construct) was supplemented with 20 μM taxol (Cytoskeleton), mixed with 2 to 3 μM MTs (tubulin; Cytoskeleton) and incubated at 37 °C. The mixture (‘input’) was layered onto 100 μl of 25% sucrose (containing 20 μM taxol, and 1 mM DTT) and centrifuged at 60,000 *g* for 10 min at 25 °C in a TLA120 rotor (Beckman). The remaining supernatant (‘S1’) was discarded and the pellet washed with 50 μl dynein ‘wash buffer’ (containing 20 μM taxol and 1 mM DTT), and then resuspended in wash buffer with 5 mM ATP (‘P1’). The solution was centrifuged again using the same parameters as above, the supernatant (‘S2’) was reserved and the pellet (‘P2’) was discarded. During the procedure, 5 μl each of input, S1, P1, S2 and P2 were reserved for SDS–PAGE analysis, as in Supplementary Fig. 7.

### Three-dimensional model rendering and figure preparation

Figures 1c,d and 2a were created with VMD^48^ and The Persistence of Vision Raytracer (POV-Ray, http://www.povray.org/) using PDB entries 3VKH, 3J2U, 1VF4 and 1IGT.

## Author contributions

P.H. and R.V. conceived the project. P.H. designed and engineered recombinant MD-WT and MD-ΔCT motor constructs, performed solution-based kinetic analysis, and with C.G.L. produced the protein. M.P.N. and A.G. designed and performed the optical trapping experiments and analyses. A.G. performed dynein-MT co-sedimentation experiments. S.B. designed and performed the MT-gliding experiments and optimized the dynein-MT co-sedimentation assay. M.P.N, S.B., P.H., R.B.V. and A.G. wrote the paper.

## Additional information

**How to cite this article:** Nicholas M. P. *et al*. Control of cytoplasmic dynein force production and processivity by its C-terminal domain. *Nat. Commun*. 6:6206 doi: 10.1038/ncomms7206 (2015).

## Supplementary Material

Supplementary Information Supplementary Figures 1-10, Supplementary Tables 1-2, Supplementary Note and Supplementary References.

Supplementary Movie 1 TMR-labeled microtubules were introduced to a slide chamber with MD-WT attached via anti-GST antibodies to the coverslip surface, and imaged in a fluorescence microscope. MD-WT drove robust gliding with an average velocity of 460 ± 80 nm/s (± SD) at 3 mM ATP. Frame rate is 7× real time.

Supplementary Movie 2 In an identical experiment to that for Supplementary Movie 1, MD-DCT did not drive MT gliding. Instead, the microtubules bind rigidly to the surface without moving, presumably due to inactive motors binding in rigor. Note the MT that lands on the surface and binds during the course of the movie. Frame rate is 7× real time. See discussion in Supplemental Information.

Supplementary Movie 3 In an identical experiment to that for Supplementary Movie 1, a yeast construct (VY149) that naturally lacks the CT-cap supports microtubule gliding with an average velocity of 120 ± 30 nm/s (± SD) at 1 mM ATP in agreement with published yeast dynein velocities, suggesting that absence of the CT-cap is not responsible for lack of gliding driven by MD-DCT (and that the assay conditions are appropriate to observe gliding generated by a motor missing the CT-cap). Frame rate is 7× real time.
