# Supplementary material for: Control of cytoplasmic dynein force production and processivity by its C-terminal domain
Source: Nat Commun. 2015 Feb 11;6:6206. doi: 10.1038/ncomms7206 (PMC4339881; doi:10.1038/ncomms7206)
Supplement: Supplementary Information — Supplementary Figures 1-10, Supplementary Tables 1-2, Supplementary Note and Supplementary References. [file ncomms7206-s1.pdf]

Supplementary Figures:

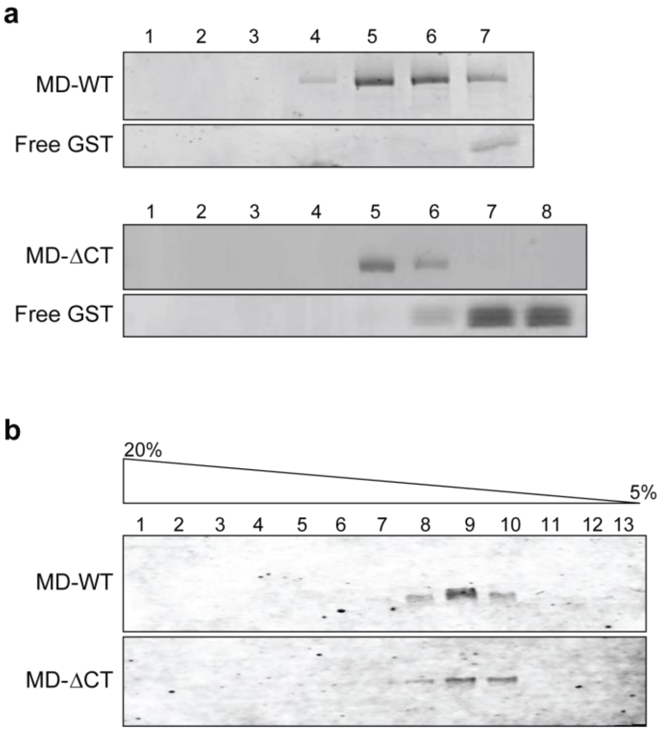

Supplementary Figure 1 | Size-exclusion chromatography purification and sucrose density gradient centrifugation. (**a**) Representative SDS-PAGE gels of size-exclusion chromatography fractionated MD-WT and MD-ΔCT preparations. Complete separation of free GST from MD-WT (fraction 5 and 6) and MD-ΔCT (fraction 5) were achieved. No evidence of larger aggregates was observed in the void volume (fraction 3). (**b**) Western blot analysis using anti-GST antibodies of the purified MD-WT and MD-ΔCT dynein after sucrose density gradient centrifugation and fractionation. Each preparation shows a single symmetric peak, indicative of a monodisperse dynein species. Faster sedimenting protein aggregates were not observed, nor was aggregated protein detected in sample from bottom of the centrifuge (fraction 1). Also, we saw no evidence for contaminating regulatory factors by Coomassie Blue staining, nor did we detect any Lis1 contamination by immunoblotting.

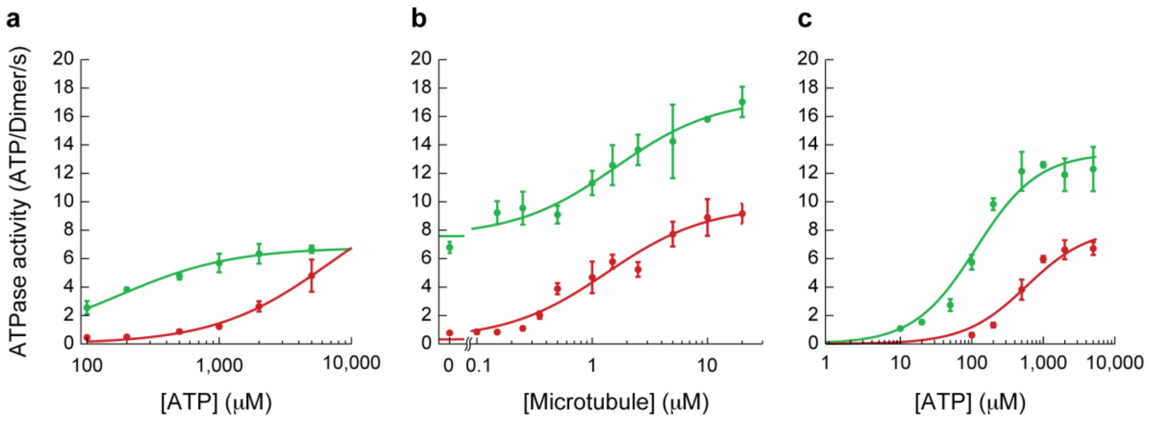

Supplementary Figure 2 | ATPase activity of MD-WT (green) and MD-ΔCT (red). (**a**) ATPase activity as a function of ATP concentration in the absence of MTs. The lines are fits using the Michaelis-Menten equation, $k_{obs} = k_{cat} \times [ATP] / (K_M(ATP) + [ATP])$. (**b**) ATPase activity as a function of MT concentration, in the presence of 1 mM ATP. The lines are fits using the equation (*3*) $k_{obs} = (k_{cat} - k_{basal}) \times [MT] / (K_M(MT) + [MT]) + k_{basal}$. (**c**) as in **a**, but in the presence of 5 μM MTs. Each point represents mean ± SD of three individual measurements. R^2 for fits to the MD-WT and MD-ΔCT data, respectively, are **a**, 0.967 and 0.965; **b**, 0.989 and 0.988; and **c**, 0.964 and 0.945. See Supplementary Table 1 and Supplemental Information for discussion.

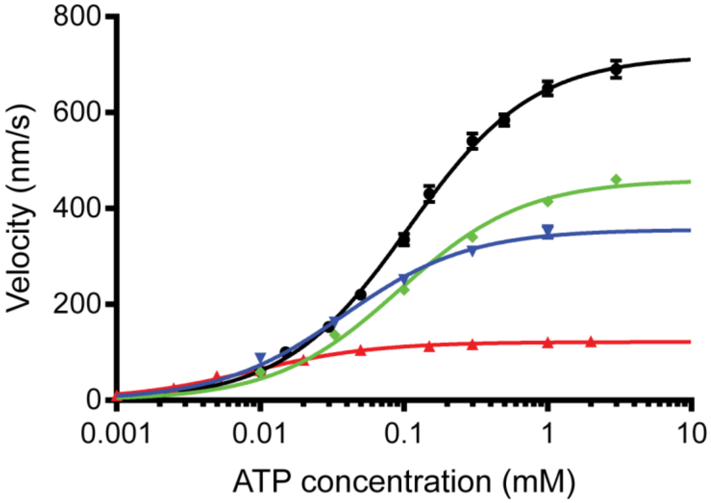

Supplementary Figure 3 | MT-gliding activity of rat dynein. Coverslips were coated with purified rat brain dynein or with anti-GST antibody, followed by MD-WT recombinant rat dynein. Results in this multi-motor assay varied by preparation and whether the protein was subjected to additional purification via size-exclusion chromatography (SEC). Lines are hyperbolic (Michaelis-Menten) fits ($v = v_{max} \times [ATP] / (K_M(ATP) + [ATP])$) yielding maximum velocities v_{max} and Michaelis-Menten constants K_M(ATP). Black circles: rat brain native dynein (v_{max} = 718 ± 7 nm/s, K_M(ATP) = 107 ± 4 µM, R^2 = 0.999). Red upward triangles: undiluted rat GST-dynein without SEC purification (v_{max} = 122 ± 1 nm/s, K_M(ATP) = 8.7 ± 0.5 µM, R^2 = 0.996). Blue downward triangles and green diamonds: undiluted rat GST-dynein from separate SEC fractions containing little (v_{max} = 356 ± 8 nm/s, K_M(ATP) = 38 ± 4 µM, R^2 = 0.996) or no (v_{max} = 460 ± 11 nm/s, K_M(ATP) = 93 ± 9 µM, R^2 = 0.995) free GST, respectively. Error bars are ±SEM of 50-300 velocity measurements. See also Supplemental Information and Supplementary Video 1.

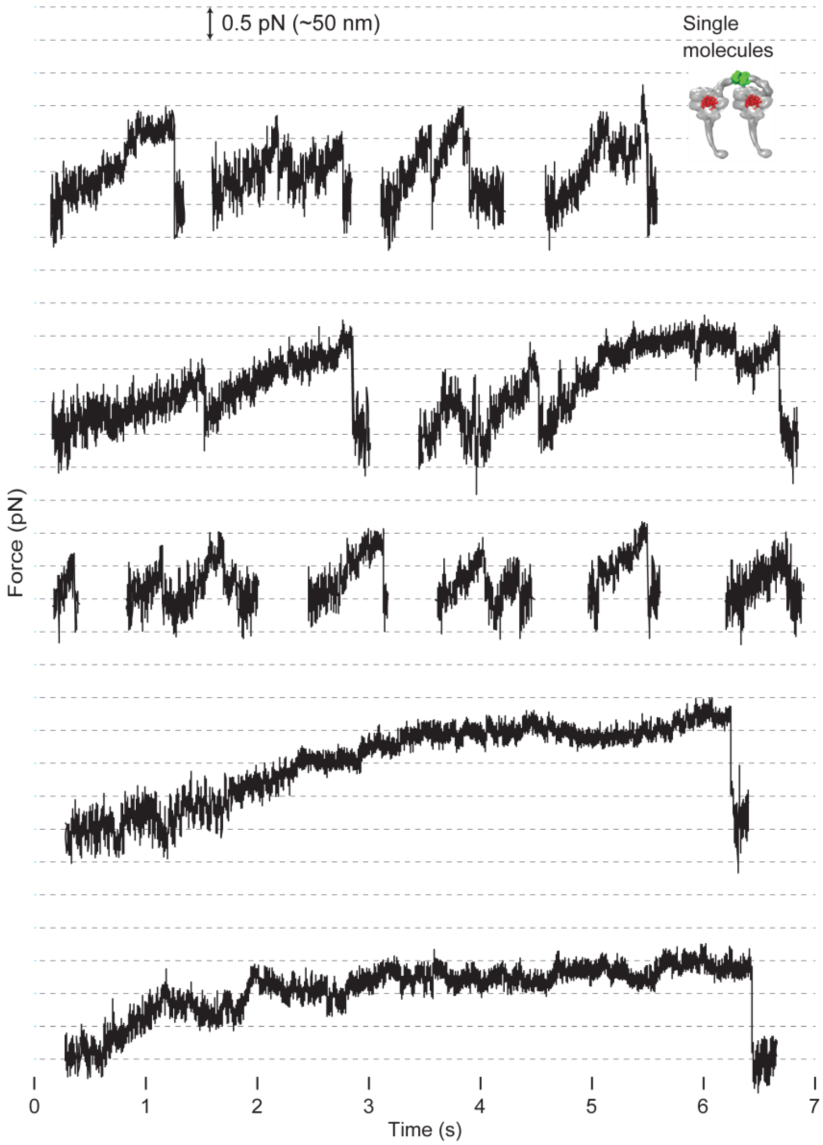

Supplementary Figure 4 | Gallery of additional representative examples of MD-WT force generation at motor concentrations for which 50% or fewer beads moved. This figure provides examples in addition to those of Fig. 2C of the main text. Prolonged stalling, as exhibited in the lower two traces, was exceedingly rare (~1-2% of events). Experiments were performed with AC-purified and AC-/SEC-purified protein.

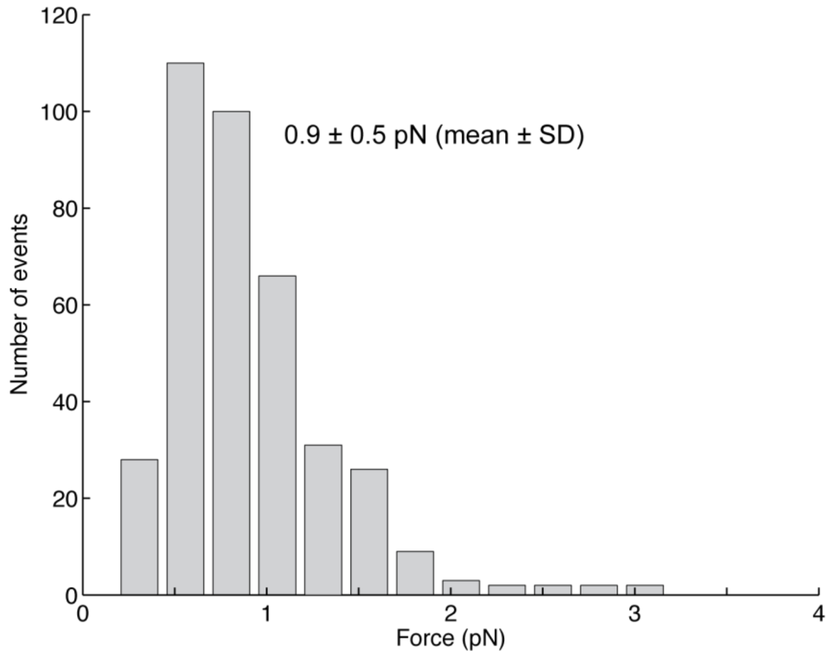

Supplementary Figure 5 | Histogram of maximal forces achieved by MD-WT during individual MT encounters. Data represent 381 events derived from 54 beads over 19 separate experiments. The spring constant used was k = 0.01 pN/nm. The mean and SD were calculated from the raw data without fitting. Experiments were performed with AC-purified and AC-/SEC-purified protein.

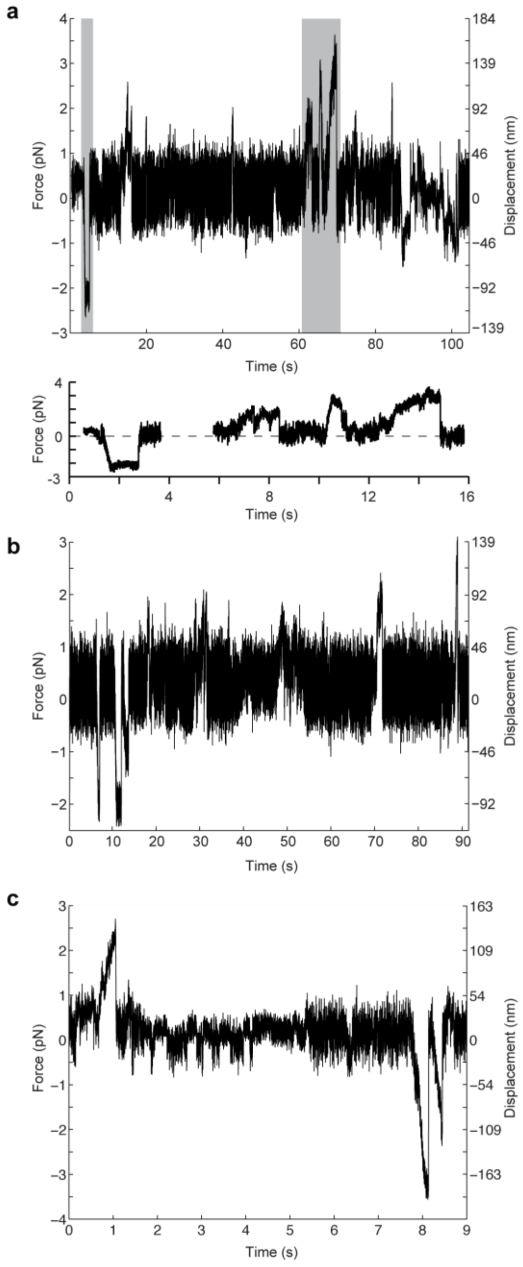

Supplementary Figure 6 | Examples of bidirectional motion at high MD-WT concentrations. (**a-c**) Example records of bidirectional force generation events at high MD-WT concentrations (several times that needed for 100% bead movement). The lower inset in **a** shows detailed views of the regions boxed in gray on the main axes. Experiments were performed with AC-purified protein.

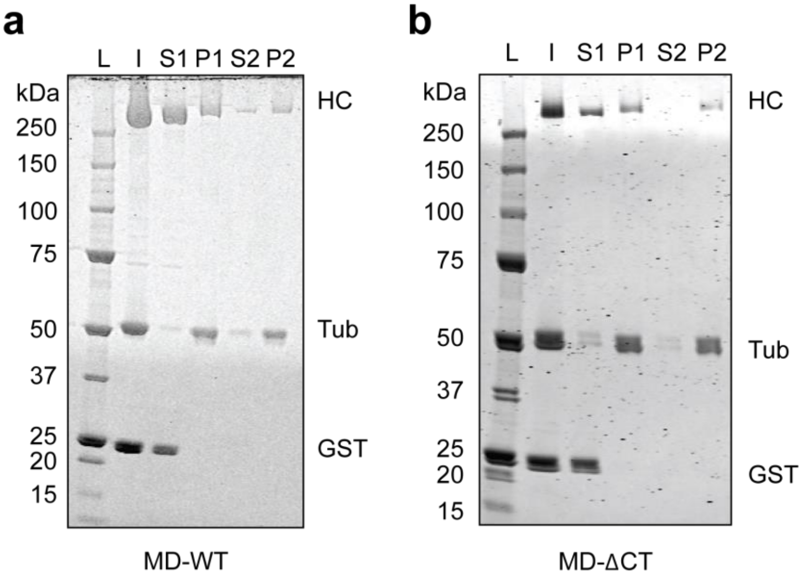

Supplementary Figure 7 | MT cosedimentation and release purification. (**a**) SDS-PAGE gel (Krypton stain) for MD-WT (360 ng/μL input). (**b**) as in **a**, but for MD-ΔCT (80 ng/μL input). Dynein was added to MTs (final tubulin concentrations: 2 μM for MD-WT and 3 μM for MD-ΔCT) and cosedimented via centrifugation (P1). After removing the supernatant (S1), the MT pellet was washed and resuspended and 5 mM ATP was added to induce motor release. The MTs were again pelleted (P2), and the supernatant (S2) was reserved. L: protein ladder, I: input, HC: dynein heavy chain, Tub: tubulin, GST: free GST. See Material and Methods and Supplemental Information for discussion.

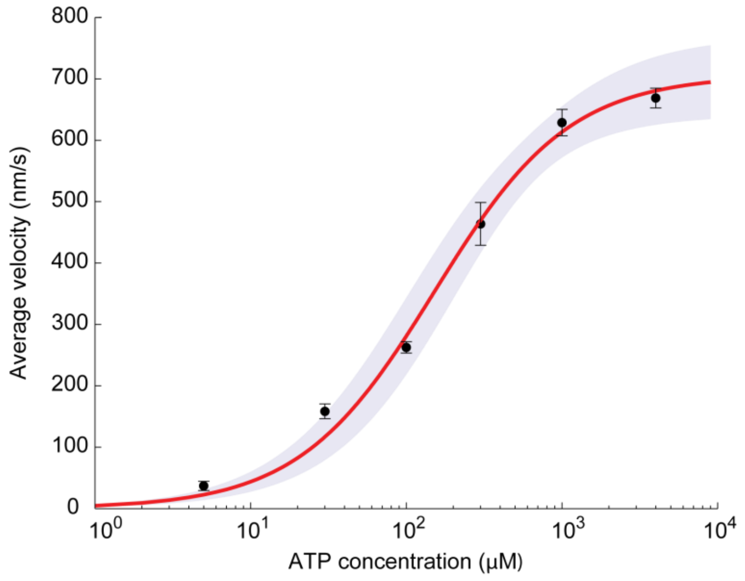

Supplementary Figure 8 | MD-ΔCT velocity under constant 0.5 pN backward load as a function of ATP concentration. Each point represents the mean of 50-230 average velocity measurements under constant 0.5 pN backward load, for runs ≥50 nm in length. Error bars are 95% CIs of the mean. The red curve is a weighted fit of the Michaelis-Menten equation $v = v_{max} \times [ATP] / ([ATP] + K_M(ATP))$ to the points, yielding v_{max} = 706 nm/s and K_M(ATP) = 151 μM (95% CI's, with Bonferroni correction, [646, 767] nm/s and [87, 216] μM, respectively). Coefficient of determination R^2 = 0.997. Since the velocity-vs.-[ATP] curve is well described by a hyperbola, our data suggest that the step size is insensitive to [ATP], assuming the underlying ATP-hydrolysis rate truly follows Michaelis-Menten kinetics (any underlying cooperative ATPase kinetics with a sigmoidal [ATP]-response would result in a velocity-vs.-[ATP] curve with even higher sigmoidicity, if the step size increased from the ~8 nm observed at low [ATP]; Supplementary Fig. 9). Experiments were performed with AC-purified protein.

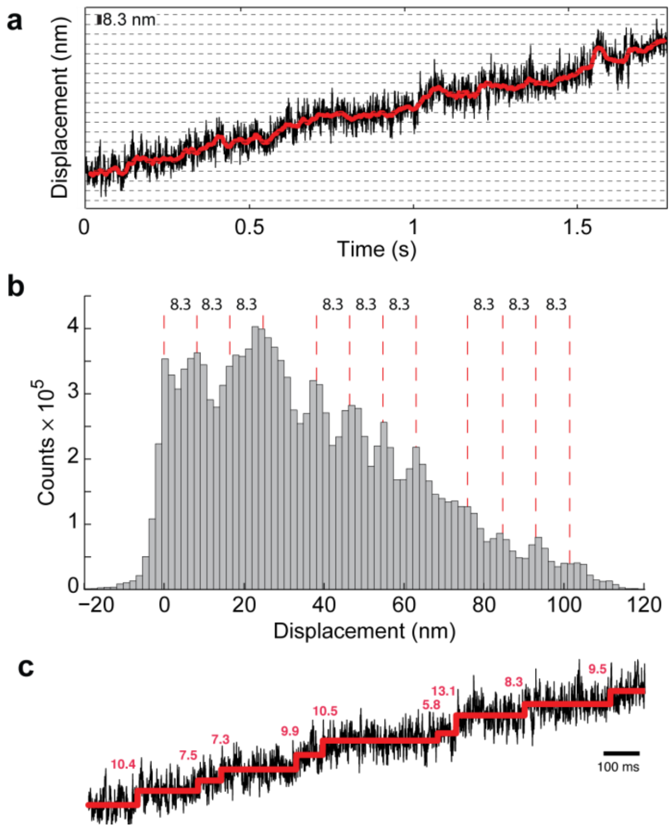

Supplementary Figure 9 | Example of MD-ΔCT stepping under 0.5 pN constant load at 5 µM ATP. (**a**) Raw data (black) and data after application of a median filter with 30 ms window (red). No steps larger than ~8 nm are resolvable by eye (trap stiffness: k = 0.031 pN/nm). (**b**) Pairwise distance distribution function (PDF) calculated from the filtered data in **a**. Groups of gridlines (red) demonstrate the predominance of ~8.3-nm separations between apparent peaks (shifting of peaks from exact 8.3 nm multiples and apparent missing peaks are likely due to instrument positional drift). (**c**) Step-size analysis of the raw data in **a** (0-1.5 s trace segment) using the step-finding algorithm developed by Kerssemakers et al. (*4*). The raw data are shown in black and the steps detected by the step-finding program in red. The analysis agrees with the predominance of ~8 nm steps as revealed by the PDF in **b**. Experiments were performed with AC-purified protein.

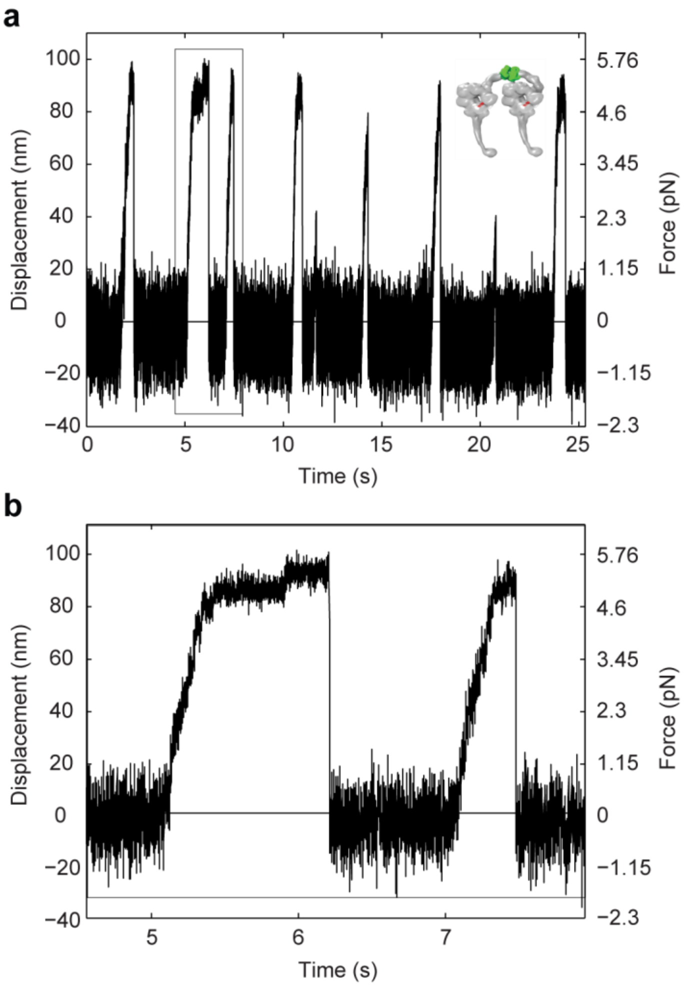

Supplementary Figure 10 | Example record of force generation by MD-ΔCT at high motor concentration. (**a**) Force/motion trace showing repeated force generation events in the trap up to ~5.5 pN (trap stiffness: k = 0.058 pN/nm) at a motor concentration at which all trapping beads tested (N = 16) either bound only or moved along MTs. (**b**) Trace segment corresponding to the trace section indicated by the rectangular box in **a** showing a motor stalling event. As the motor approaches stalling, the velocity slows considerably, and ~8-nm steps are clearly resolvable. Experiments were performed with AC-purified protein.

Supplementary Tables:

	Basal ATPase (No MTs)		MT-Stimulated ATPase				
	**k_{cat} (s^{-1})**	**K_M(ATP) (μM)**	**k_{basal} (s^{-1})**	**k_{cat}' (s^{-1})**	**K_M(MT) (μM)**	**k_{cat}'' (s^{-1})**	**K_M(ATP)' (μM)**
**MD-WT**	6.8 ± 0.1	172 ± 16	7.6 ± 0.5	17.3 ± 0.7	1.60 ± 0.46	13.3 ± 0.7	114 ± 24
**MD-ΔCT**	11 ± 2	6,800 ± 2,000	0.31 ± 0.4	9.7 ± 0.6	1.34 ± 0.36	8.2 ± 0.9	587 ± 200

Supplementary Table 1 | Summary of ATPase activity (per dimer). Values are given from the fits in Supplementary Fig. 2, ± standard errors of the fit values. k_{cat} is the maximal ATPase rate in the absence of MTs and K_M(ATP) is the ATP concentration producing a rate of ½k_{cat} in the absence of MTs (see Supplementary Fig. 2A). $k_{basal,}$ k_{cat}', and K_M(MT) were all measured in the presence of 1 mM ATP (see Supplementary Fig. 2B). Thus, k_{basal} is the ATPase rate in the absence of MTs for this particular ATP concentration; k_{cat}' maximally MT-stimulated rate at 1 mM ATP, and K_M(MT) is the MT concentration yielding ½k_{cat}'. k_{cat}'' and K_M(ATP)' are analogous to k_{cat} and K_M(ATP), respectively, except that 5 μM MTs were present (see Supplementary Fig. 2C). See Supplemental Information for discussion.

**K_M(ATP) (μM)**	**Species**	**MT conc. (μM)**	**Reference**
15	Cow	18	Shpetner et al. (*5*)
33 or 280*	Cow	NA (MT gliding)	Shimizu et al. (*6*)
89	Pig	NA (optical trapping)	Toba et al. (*7*)
27	Mouse	10	Ori-McKenney et al (*8*)
18	*D. discoideum*	10	Kon et al. (*9*)
26	*S. Cerevisiae*	5	Cho et al. (*1*)

Supplementary Table 2: Reported K_M(ATP) values for cytoplasmic dynein. NA: not applicable. *Different methods of calculation yielded different results.

Supplementary Note:

Notes on bidirectional motility observed at high motor concentration

Bidirectional motion was observed only at very high motor concentrations (100% of beads exhibiting motion), and never at the single-molecule level (≤50% beads exhibiting motion). The behavior occurred on multiple MTs and also on axonemes. However, it was not observed for every bead tested under these conditions, and backward movement was often seen only sporadically. This may indicate that reversal of movement only occurs for specific configurations of grouped motors. Kinesin contamination was highly unlikely given the expression and purification strategies, as well as the specific linkage of the motor to the bead via an anti-GST antibody. Careful visual inspection of the fluorescence-labeled MTs that supported bidirectional motion never revealed any evidence of MT bundling that might lead to antiparallel tracks for the motors to move upon.

Bidirectional movement was only observed once for MD-ΔCT (again, at very high motor concentration). This may reflect an inherent difference between MD-WT and MD-ΔCT. However, we believe it is more likely explained by the relatively low fraction of active MD-ΔCT motors in our preparations (see below), which would decrease the likelihood of active motors attaching near each other on the trapping bead surface. Consistent with this, even at relatively high motor concentrations, the behavior of MD-ΔCT (e.g. velocity and stall force) was not noticeably changed from that at the single-molecule level, suggesting that even at high concentrations, single motors drove the vast majority of events (Supplementary Fig. 10).

Notes on the proportions of active dynein motors

Our purified MD-WT and MD-ΔCT samples were well behaved as judged by the absence of degradation and aggregation (Supplementary Fig. 1). However, multiple observations suggest that the MD-ΔCT construct is particularly vulnerable to loss of activity. The enzymatic rate k_{cat} obtained from ATPase assays at saturating MT concentration was variable from one preparation to the next, ranging from ~13 ATP/s to ~17 ATP/s for MD-WT and ~1 ATP/s to ~9 ATP/s for MD-ΔCT (the lower ATPase activities of MD-ΔCT might in part reflect the very high basal and MT-activated K_M(ATP) values for this construct determined enzymatically, see Supplementary Table 1). Low measured ATPase activity correlated with a low fraction of motile beads in the optical trapping assay (even at high motor concentrations) and some preparations of both constructs failed to produce any motility. This was probably due to inactive motors occupying the majority of the binding sites on most beads. However, the contribution of preparative conditions, freeze/thaw, and freshness to protein stability remains incompletely resolved.

Unlike MD-WT (Supplementary Fig. 3 and Supplementary Video 1), MD-ΔCT did not support MT gliding, and instead MTs bound rigidly to the motor-coated surface (Supplementary Video 2), even when diluted 10,000× from starting concentration. Similarly, when attempting further purification via cosedimentation with MTs followed by ATP-induced release, no detectable MD-ΔCT released from MTs, even in the presence of 5 mM ATP (Supplementary Fig. 7B). These findings might suggest that MD-ΔCT is immotile, has a slow enzymatic rate, or has a very high affinity for MTs (even in the presence of 5 mM ATP and 200 mM KCl; this behavior is partly consistent with that of yeast MD dimers, which can be extracted with inclusion of high salt, though this still had no apparent effect on rat MD-ΔCT extraction). However, the robust motility observed in the trap suggests that single MD-ΔCT motors are highly active. Indeed, we measured

the maximal velocity for the motor under low load to be ~700 nm/s (Fig. 3E and Supplementary Fig. 8). Given the 8-nm step size, this implies $k_{cat} \approx 90\ s^{-1}$, which is at least 10-fold greater than the result calculated from MT-stimulated ATPase measurements. This could mean that less than 10% of the motors in such a sample are active depending on the preparation. It is also notable that, under identical experimental conditions, a GST-dimerized yeast dynein analogous to MD-ΔCT supported robust MT gliding (Supplementary Video 3). Our optical trapping analysis of MT interactions of motor-coated beads further supports the conclusion that a large percentage of the MD-ΔCT motors is inactive. While trapping beads without bound motors did not show any MT interactions, beads incubated with motors either generated force and displacements or bound to MTs without detectable force-generation events. In contrast to beads coated with MD-WT motors, of which 90-100% of all MT-interacting beads generated forces above the detection limit of 0.2 pN, less than 20% of the MT-interacting beads coated with MD-ΔCT motors showed force-generation (the remaining MT-interacting beads coated with MD-ΔCT motors bound strongly to MTs without detectable force generation). To ensure single-molecule MT-interactions of either inactive or active motor molecules, these analyses were performed at motor concentrations where less than 30% of all beads tested interacted with MTs. This further supports the idea that a large fraction of inactive motors, rather than poor enzyme activity, is responsible for the results from the ensemble (ATPase and MT gliding) assays.

Given these challenges, we opted to avoid detailed mechanochemical interpretation of ensemble assays for MD-WT and MD-ΔCT, and we instead focused on unambiguous single-molecule measurements using the optical trap. It is notable that both MD-WT and MD-ΔCT retained activity in this assay up to multiple hours, suggesting that the observed loss of activity may result

from failure to properly fold during expression, damage experienced during subsequent purification steps, or denaturation during freezing/thawing of stored aliquots. This may also reflect improved enzyme stability when attached to the trapping beads. Alternatively, it is conceivable that individual motors transition between active and inactive states, spending most of their time in the latter.

Discussion of mechanisms for regulation of dynein force and processivity by the CT-cap

Considerable future work will be needed to define the precise mechanism by which removal of the CT-cap from the mammalian dynein motor domain (MD) imparts it with yeast dynein-like force production and processivity. As mentioned in the main text, one possibility is that the CT-cap controls nucleotide affinity of the AAA1 active site via direct interactions. This way the CT-cap could control force-bearing states of the dynein cross-bridge cycle and make the motor less able to withstand opposing load and more likely to release after a few steps. Some of our ATPase and MT-gliding assay results suggested a larger K_M(ATP) for MD-ΔCT than for MD-WT, which could indicate a difference in nucleotide affinity. However, both ensemble techniques yielded variable results depending on the protein preparation assayed. In the MT-gliding assay, in addition to variations among different preparations, the measured kinetics differed within individual preparations depending on which fraction from size exclusion chromatography was used (Supplementary Fig. 3), possibly due to assay-specific effects arising from different amounts of GST competing with the GST-tagged dynein for surface-bound anti-GST antibodies on the glass.

Single-molecule function was reproducible and far easier to interpret. MD-WT showed indistinguishable behavior on the single-molecule level whether GST fragments were present or not. Due to its limited processivity and low stall force, characterization of MD-WT K_M(ATP) at low load was infeasible. However, we were able to characterize the K_M(ATP) for MD-ΔCT (Supplementary Fig. 8) at low load (0.5 pN), yielding K_M(ATP) = 151 μM (95% CI [87, 216] μM). Comparing this K_M(ATP) to values reported previously (as well as values obtained in the gliding assay for both GST-dimerized and native dynein) does not suggest a substantial difference from higher eukaryotic wild-type dyneins (although the reported values span a fairly large range) (Supplementary Table 2). Moreover, the K_M(ATP) reported for yeast dynein (*1*) (Supplementary Table 2) is nearly as small as the lowest reported values for higher eukaryotes, suggesting that removal of the CT-cap does not, by itself, drastically affect K_M(ATP). Nonetheless, the dramatic decrease in vanadate sensitivity caused by removal of the CT-cap (*2*) suggests that some step in the catalytic cycle is under the control of dynein's C-terminal domain. In conclusion, future work is needed to determine whether the CT-cap alters AAA1 function directly or acts as a "mechanical" element that affects the allosteric communication between the AAA ring and the MTBD.

Supplementary References

1. C. Cho, S. L. Reck-Peterson, R. D. Vale, Regulatory ATPase sites of cytoplasmic dynein affect processivity and force generation. *J. Biol. Chem.* **283**, 25839-25845 (2008).
2. P. Höök, A. Mikami, B. Shafer, B. T. Chait, S. S. Rosenfeld, R. B. Vallee, Long range allosteric control of cytoplasmic dynein ATPase activity by the stalk and C-terminal domains. *J. Biol. Chem*. **280**, 33045-33054 (2005).
3. M. Nishiura, T. Kon, K. Shiroguchi, R. Ohkura, T. Shima, Y. Y. Toyoshima, K. Sutoh, A single-headed recombinant fragment of Dictyostelium cytoplasmic dynein can drive the robust sliding of microtubules. *J. Biol. Chem*. **279**, 22799-22802 (2004).
4. J. W. J. Kerssemakers, E. L. Munteanu, L. Laan, T. L. Noetzel, M. E. Janson, M. Dogterom, Assembly dynamics of microtubules at molecular resolution. *Nature* 442, 709-712 (2006).
5. H. S. Shpetner, B. M. Paschal, R. B. Vallee, Characterization of the microtubule-activated ATPase of brain cytoplasmic dynein (MAP 1C). *J. Cell Biol*. **107**, 1001-1009 (1988).
6. T. Shimizu, Y. Y. Toyoshima, M. Edamatsu, R. D. Vale, Comparison of the motile and enzymatic properties of two microtubule minus-end-directed motors, ncd and cytoplasmic dynein. *Biochemistry* **34**, 1575-1582 (1995).
7. S. Toba, T. M. Watanabe, L. Yamaguchi-Okimoto, Y. Y. Toyoshima, H. Higuchi, Overlapping hand-over-hand mechanism of single molecular motility of cytoplasmic dynein. *Proc Natl Acad Sci U S A* **103**, 5741-5745 (2006).
8. K. M. Ori-McKenney, J. Xu, S. P. Gross, R. B. Vallee, A cytoplasmic dynein tail mutation impairs motor processivity. *Nat Cell Biol* **12**, 1228-1234 (2010).
9. T. Kon, M. Nishiura, R. Ohkura, Y. Y. Toyoshima, K. Sutoh, Distinct functions of nucleotide-binding/hydrolysis sites in the four AAA modules of cytoplasmic dynein. *Biochemistry* **43**, 11266-11274 (2004).
